# A Review of Controversial Issues in the Management of Head and Neck Cancer: A Swiss Multidisciplinary and Multi-Institutional Patterns of Care Study—Part 1 (Head and Neck Surgery)

**DOI:** 10.3389/fonc.2019.01125

**Published:** 2019-10-24

**Authors:** Pavel Dulguerov, Martina A. Broglie, Guido Henke, Marco Siano, Paul Martin Putora, Christian Simon, Daniel Zwahlen, Gerhard F. Huber, Giorgio Ballerini, Lorenza Beffa, Roland Giger, Sacha Rothschild, Sandro V. Negri, Olgun Elicin

**Affiliations:** ^1^Department of Otorhinolaryngology, Head and Neck Surgery, Geneva University Hospital, Geneva, Switzerland; ^2^Department of Otorhinolaryngology, Head and Neck Surgery, Cantonal Hospital St. Gallen, St. Gallen, Switzerland; ^3^Department of Otorhinolaryngology, Head and Neck Surgery, University Hospital Zurich, Zurich, Switzerland; ^4^Department of Radiation Oncology, Cantonal Hospital St. Gallen, St. Gallen, Switzerland; ^5^Department of Medical Oncology, Cantonal Hospital St. Gallen, St. Gallen, Switzerland; ^6^Department of Medical Oncology, Hôpital Riviera-Chablais, Vevey, Switzerland; ^7^Department of Radiation Oncology, Inselspital, Bern University Hospital, University of Bern, Bern, Switzerland; ^8^Department of Otorhinolaryngology, Head and Neck Surgery, University Hospital of Lausanne, Lausanne, Switzerland; ^9^Department of Radiation Oncology, Cantonal Hospital Graubünden, Chur, Switzerland; ^10^Department of Radiation Oncology, Cantonal Hospital of Winterthur, Winterthur, Switzerland; ^11^Department of Radiation Oncology, Clinica Luganese SA, Lugano, Switzerland; ^12^Department of Radiation Oncology, Cantonal Hospital Lucerne, Lucerne, Switzerland; ^13^Department of Otorhinolaryngology, Head and Neck Surgery, Inselspital, Bern University Hospital, Bern, Switzerland; ^14^Department of Medical Oncology, University Hospital of Basel, Basel, Switzerland; ^15^Department of Otorhinolaryngology, Lindenhofspital, Bern, Switzerland

**Keywords:** consensus, head and neck cancer, patterns of care, practice patterns, survey

## Abstract

**Background:** The Head and Neck Cancer Working Group of Swiss Group for Clinical Cancer Research (SAKK) has investigated the level of consensus (LOC) and discrepancy in everyday practice of diagnosis and treatment in head and neck cancer.

**Materials and Methods:** An online survey was iteratively generated with 10 Swiss university and teaching hospitals. LOC below 50% was defined as no agreement, while higher LOC were arbitrarily categorized as low (51–74%), moderate (75–84%), and high (≥85%).

**Results:** Any LOC was achieved in 62% of topics (*n* = 60). High, moderate and low LOC were found in 18, 20, and 23%, respectively. Regarding Head and Neck Surgery, Radiation Oncology, Medical Oncology, and biomarkers, LOC was achieved in 50, 57, 83, and 43%, respectively.

**Conclusions:** Consensus on clinical topics is rather low for surgeons and radiation oncologists. The questions discussed might highlight discrepancies, stimulate standardization of practice, and prioritize topics for future clinical research.

## Introduction

The cause of heterogeneity in the practice of diagnosis and treatment of head and neck squamous cell carcinoma (HNSCC) can be associated with multiple factors: differences in health care policies, financial and logistic factors, variations in tradition and medical culture between geographical areas, institutions, or even among physicians working in the same hospital. This heterogeneity in patterns of care is expected to be inversely correlated with the level of evidence on a given topic.

Literature on various aspects of management of HNSCC were previously published. Such reports usually focused on an anatomical site of the head and neck area (1, 2), a specific treatment approach in a clinical discipline (3–6), diagnostic modalities and strategies for diagnosis (7) and follow-up (8). Most of these survey-based studies were performed among institutions sharing the same geography or language.

The Head and Neck Cancer Working Group of Swiss Group for Clinical Cancer Research (SAKK) is a multidisciplinary collective of head and neck cancer specialists from many Swiss institutions meeting in regular intervals and collaborating in various projects. Due to the lack of a similar comprehensive work published so far, the group decided to perform a survey covering a broad spectrum of controversial topics concerning the diagnosis and the treatment of HNSCC among its member institutions.

This survey was designed to discuss current diagnostic and treatment strategies for HNSCC of all localizations undergoing within the Head and Neck Cancer Working Group of SAKK (multidisciplinary and multi-institutional) and to find out probable differences between the participating members/institutions in a pattern of care study.

## Materials and Methods

In order to investigate the consensus and heterogeneity in the various aspects of diagnosis and treatment of HNSCC, an online survey via Surveymonkey® (San Mateo, CA) was generated and used by taking the following steps.

1. A steering committee of two head and neck surgeons, one medical oncologist and three radiation oncologists (P-M. P. serving as a consultant for methodology and technical issues) was founded to generate a questionnaire draft, evaluate the answers and writing the final manuscript.
2. Centers in which every patient diagnosed with a HNSCC is presented and discussed on a multidisciplinary tumor board on a regular basis, were defined and contacted through the member list of SAKK by email or phone and a local coordinator for each center was assigned. A rather balanced distribution of the specialists defined as local coordinators from the disciplines of head and neck surgery, medical oncology, and radiation oncology was encouraged. The responsibility of the local coordinator (e.g., the medical oncologist in a center) was to address the part of the questionnaire related to their specialty (medical oncology) and organize the information flow with her/his institutional colleagues from the remaining two major disciplines (head and neck surgery and radiation oncology). As a trade-off between being completely inclusive and realistically conducting the survey, specialists of the above-mentioned three disciplines were asked also to address the questions about imaging, pathology, and maxillo-facial surgery on behalf of the corresponding specialists of these disciplines.
3. The preliminary draft of the questionnaire was generated by the steering committee and sent to each local coordinator. Four categories were generated: head and neck surgery, radiation oncology, medical oncology, and biomarkers. Each center was asked to assign a numerical point for each question, proportionally reflecting its level of importance in the concerning category. Centers were also asked for feedback for any unclear questions, to suggest modifications and new questions.
4. After receiving feedbacks about the draft version, the questionnaire was finalized for improved wording as suggested and based on two criteria: (1) if a new question was suggested from more than one center in same or similar context, it was added to the final version, and (2) each category was limited with a maximal number of 20 questions, and questions with lower cumulative points were eliminated.
5. The final version (Supplementary Material) was transformed into an online survey and each center was asked to fill out the questionnaire. Each center is represented by a local coordinator as listed in the co-authors and their affiliations.
6. Answers were evaluated and discussed by the steering committee. Similar topics were grouped together.
7. For each question, an agreement per center is counted as 10 and a disagreement as 0, giving a minimal score of 0 and a maximal score of 100. Missing answers are indicated in the corresponding denominators. Level of consensus (LOC) was calculated by summing all center's answers and categorized as lack of LOC (0–50%), low LOC (51–74%), moderate (75–84%), and high (≥85%).

## Results and Discussion

Ten centers participated in the survey. The survey was completed on 13 September 2017. Possible practice changes which may have occurred after this date were not reflected in this manuscript. Union for International Cancer Control (UICC) 7th edition (9) was used for discussions related to staging.

Some LOC was achieved in 62% of all topics of interest, while no LOC was found in 38% of questions. High, moderate and low LOC were 18, 20, and 23%, respectively. LOC in each section is summarized in Table 1.

**Table 1:** Level of consensus in each section.

**Section**	**Overall (>50%)**	**Low (51–74%)**	**Moderate (75–84%)**	**High (85–100%)**
Head and neck surgery	50%	21%	0%	29%
Radiation oncology	57%	14%	33%	10%
Medical oncology	83%	39%	22%	22%
Biomarkers	43%	14%	14%	14%

Following section provides the results for the items concerning head and neck surgery discipline, each followed by a short discussion if deemed relevant.

### Head and Neck Surgery

#### Diagnostic Measures

➢ *Routine use of diagnostic panendoscopy: high LOC (100%)*.

During the diagnosis and baseline workup, all (10/10) centers routinely performed an endoscopy of the upper aerodigestive tract under general anesthesia to detect synchronous secondary malignancies. In one center, panendoscopy was not part of the routine workup for patients without history of tobacco or alcohol abuse.

The incidence of synchronous HNSCC around 5–6% (10, 11) is considered high enough to require a diagnostic panendoscopy. Usually, the second primary is of small size and thus curable. Hence, the diagnosis of synchronous lesions usually alters the therapeutic approach. Since ^18^F-fluorodeoxyglucose positron emission tomography combined with computerized tomography (^18^FDG-PET/CT) is often performed during the evaluation or treatment planning, some have suggested that a ^18^FDG-PET/CT scan could replace endoscopy (12). However, ^18^FDG-PET/CT will not detect small superficial lesions which are main focus of endoscopy (13, 14) and Swiss centers are unanimous in using panendoscopy during the initial evaluation. However, this practice can be questioned in non-smoker patients who are diagnosed with a Human Papillomavirus (HPV)-associated oropharyngeal squamous cell carcinoma (OPSCC) due to the decreased rates of secondary malignancies (15–17).

➢ *Routine diagnostic use of* ^*18*^*FDG-PET/CT to address loco-regional extension: low LOC (60%)*.
➢ *Routine diagnostic use of* ^*18*^*FDG-PET/CT to address distant metastases or second primary tumors is preferred: high LOC (100%)*.

The use of ^18^FDG-PET/CT for the purpose of determining the extent of the loco-regional disease is being used in 6/10 centers. In all centers ^18^FDG-PET/CT was undergone to detect/rule out distant metastases or locate the primary tumor in the staging of a clinical carcinoma of unknown primary (CUP).

There is no high-level evidence for or against the value of the ^18^FDG-PET/CT for an accurate estimation of the extent of the disease, especially for the primary site. Since the gold standard is the assessment of the surgical specimen, a correlation between parameters such as dimensions, volume, depth, or involvement of critical structures obtained radiologically and pathologically is sought (18). Because of the distortions and shrinkage of surgical specimen, few studies have been undertaken especially for ^18^FDG-PET/CT. The available data for ^18^FDG-PET/CT is restricted to laryngo-hypopharyngeal primaries and is based on a total of 19 patients (19, 20): tumor volume estimation seems accurate but the superficial extension was inaccurate. While surgeons possibly have the direct estimation of the superficial spread to complement the radiologic findings, the widespread use of ^18^FDG-PET/CT on target volume delineation for radiation could be questioned.

In some series, the sensitivity of ^18^FDG-PET/CT is shown to be superior to CT and MRI for the identification of occult neck lymph node metastases (21). However, the sensibility of all techniques remains low in this setting, around 60% (22). ^18^FDG-PET/CT seems to accurately estimate volumes of metastatic neck lymph nodes (23), but adds marginal value to the information obtained from standard imaging modalities, such as CT or MRI in clinically N+ patients (24).

The role of ^18^FDG-PET/CT for the diagnosis of distant metastases seems more straightforward, but because of the low incidence of distant metastases from HNSCC at initial presentation, it should be restricted to advanced N stages. Furthermore, ^18^FDG-PET/CT is useful to diagnose synchronous cancers such as lung or abdominal primaries, although the superiority over chest CT has not been demonstrated (25).

For unknown primaries, the added diagnostic value of ^18^FDG-PET/CT in the pre-HPV era was about 20% (26), while small recent studies and imaging modalities might increase the yield to 50% (27).

#### Management of the Neck

➢*Use of sentinel lymph node biopsy (SLNB) in cN0 oral cavity tumors: no consensus*.

In cN0 oral cavity primaries, SLNB is performed only in 4/10 centers. The reasons not to perform the technique were not queried.

The only randomized prospective study in cN0 oral cavity management concluded that neck exploration during the initial treatment resulted in better overall and disease-free survival than observation followed by therapeutic neck dissection for nodal recurrences. This study validated elective neck dissection, not sentinel neck biopsy (28).

Proponents of SLNB in cN0 neck stress that many patients (70%) will have a non-metastatic neck and therefore will be overtreated by a surgery associated with a substantial morbidity. If this line of arguments is followed, omitting SLNB in oral cavity primaries could be seen as suboptimal surgical oncology management.

The arguments against a SLNB approach when comparing it to the traditional elective neck dissection include: (1) oncologic inferiority, (2) unavailability or unreliability of frozen sections in SLNB, (3) need of a second procedure in case of SLNB positivity, (4) technical challenges and learning curve of the procedure, (5) lack of conviction in the difference in morbidity between the two approaches. The arguments for a SLNB include (1) less invasive approach, (2) second stage completion neck dissection only necessary in the minority of patients (25–30%), (3) selective detection of the lymph nodes of highest risk to harbor metastatic disease, (4) the pathologic workup of sentinel lymph nodes allows for the detection of small metastatic disease such as isolated tumor cells and micrometastases rather than macrometastases only leading to a more accurate staging of the neck.

Because of the pathology processing, most pathologists are reluctant to recommend frozen sections in a sentinel lymph node approach. Since frozen section of a sentinel lymph node usually consists in the examination of a single section, several studies have found this technique is suboptimal or unreliable (29). The unavailability of frozen sections or their lack of reliability makes most centers use SLNB during one procedure, with a subsequent neck dissection performed during a second operation. If the initial panendoscopy is performed as a separate procedure, this could make three general anesthesia for the treatment of a T1 carcinoma.

An elective neck dissection approach with frozen sections of lymph nodes appearing suspicious during the procedure allows for definitive neck management by completing the dissection in the same surgical setting (therapeutic neck dissection) when frozen section yields occult nodal metastasis.

The advocates of SLNB consider that 20–50 cases are necessary during the learning phase of the technique, while most head and neck surgeons dealing with cancer are quite proficient in elective neck dissection. Other problems include the necessity of a nuclear medicine exam, the necessity of the surgeon to be available for the intraoral injection, the pain associated with the awake intraoral injection, and difficulties of scheduling an operating theater with a specific delay after the injection.

Beyond difficulties accepting new techniques, if the morbidity associated with elective supra-omohyoid selective neck dissection was considerable, oncologic head and neck surgeons would have had adopted SLNB readily. However, around half of the centers probably consider that convincing data of such superiority is lacking (30, 31). Probably the main advantage of SLNB is the more thorough pathologic examination of the lymph nodes most at risk, but the exact oncologic significance of micro-metastasis in HNSCC remains to be determined.

Whether, an N0 neck is treated by elective neck dissection or SLNB, follow-up is essential, especially for necks not requiring adjuvant therapy. Radiologic surveillance could be accomplished by various modalities (CT, MRI, and US) with US-FNAC being the most accurate and cost-effective (32, 33). This neck follow-up policy is valid in other situations where the primary is treated surgically and the neck not treated, for example an early laryngeal primary.

➢*Standard use of any up-front neck dissection strategy for advanced neck stages: no consensus*.

In the chemoradiotherapy (CRT) setting, 4/8 centers pursue a systematic elective neck dissection strategy. Three of those 4 perform an up-front neck dissection in case of a cN2/3 disease, whereas a planned neck dissection 8–12 weeks after CRT is preferred in the fourth center.

CRT has become the preferred strategy for pharyngeal (34) and laryngeal (35) primaries in some centers. Advanced stage disease is often associated with bulky (N3) or multiple (N2b/c, N3) neck lymph node metastasis and the optimal strategy to treat these metastatic neck diseases remains controversial. Possible strategies include: (1) up-front neck dissection before CRT; (2) planned neck dissection after CRT; or (3) radiologic surveillance. Several Swiss centers have pursued the up-front neck dissection since the 1990's (36, 37) and have not found convincing arguments to change their strategy (38). Until recently, the debate has been centered on whether a planned neck dissection after CRT is necessary and whether a post-treatment ^18^FDG-PET/CT scan can be used to select patients needing surgery. This has been settled in a randomized controlled trial showing that a post-treatment ^18^FDG-PET/CT scan would safely identify patients not requiring neck dissection after CRT (39). The question of up-front neck dissection vs. post-CRT treatment is the subject of an ongoing prospective multicenter study in Switzerland (NCT02918955).

➢*Systematic division and reporting of lymph node levels after a neck dissection: no consensus*.

When performing a neck dissection, 4/8 centers systematically mark the lymph node stations before sending the material to pathology.

Whether neck dissection is therapeutic (cN+) or elective (cN0), one of its main purposes is to determine which patients are candidates for adjuvant therapy (40). Since neck irradiation is no longer performed by lateral opposed fields but by intensity modulated radiotherapy optimized via inverse planning, precise knowledge of the metastatic groups is crucial to the radiation oncologist. The American Head & Neck Society recommends that neck contents should be divided into levels and sublevels by the surgeon in the operating room immediately after the specimen is removed, each level being placed into a separate container and labeled appropriately (41, 42). One possible exception to these guidelines is obtaining negative margins on bulky and obviously metastatic nodes, which might require keeping two or three adjacent levels together. Even a pathologist specialized in HNSCC has trouble deciding on the limits of individual groups without the orienting presence of the hyoid bone and of the cricoid cartilage, especially on a neck dissection specimen fixed in formalin.

➢*Impact of depth of tongue infiltration on the decision to perform a neck dissection: no consensus*.

For the carcinoma of the lateral side of the tongue, the depth of invasion does not influence the decision to perform a neck dissection in two centers. In five centers, 2–8 mm depth of invasion (mean and median 4 mm) would change the treatment strategy. Three centers did not provide any answer.

Convincing data on the relationship between tumor thickness and prognosis in oral cavity squamous cell carcinoma date back to the 1980's (43). Recently and after this questionnaire was completed, depth of invasion was incorporated in the T staging system for oral cavity carcinoma and validated in recent studies (44, 45). The treatment strategy, especially for neck management, should be more aggressive with depth of invasion >4 mm (44, 46).

#### Management of Bone and Peri-Neural Invasion

➢*Adequate resection margin of mandible in case of bone invasion: no consensus*.

In case the CT and/or MRI suggest a 2 cm long cortical defect on the body of the mandible with a 5 mm depth of invasion without any enhancement of the mandibular nerve, resection margins of 1, 2, and 3 centimeters would be used in 3, 2, and 1 centers, respectively. Four centers did not provide any answer.

Three decades ago, Slootweg and Muller (47) described two patterns of mandibular invasion: an “erosive pattern” carrying a good prognosis and associated with direct bone infiltration by the carcinoma, on a broad front, without infiltration of the periodontal ligament and of the inferior alveolar nerve. The “infiltrative pattern” carries a worse prognosis and histologically exhibits an aggressive invasion of mandibular cancellous marrow, periodontal ligament, as well as a frequent perineural invasion of the inferior alveolar nerve. Subsequent series (48, 49) have confirmed two- to three-fold higher recurrence rates and approximately halved survival in the infiltrative pattern of invasion. Furthermore, because cortical bone invasion does not carry a poor prognosis, it has been suggested that to stage it as T3 (50).

The literature rarely speaks of “erosive” and “infiltrative” pattern but often refers to cortical vs. marrow infiltration. Preoperative performance for mandibular marrow invasion of MRI carries a high sensitivity (95–100%) but a lower specificity (60–70%) (51, 52).

According to the Dutch Guidelines Database (53), in the erosive pattern a bony margin of 1 cm is sufficient, while the infiltrative pattern requires bony margins of 1.5 cm and invasion within the canal of the mandibular nerve 2 cm. While these recommendations are cited in the literature, their exact scientific foundation is unclear.

➢*The indication to perform a mandibulectomy in case of mandibular nerve invasion: no consensus*.

In case of an oral cavity tumor where the MRI suggests an enhancement of the mandibular nerve and the CT shows no erosion of the mandible, 2/8 centers would perform a mandibulectomy, whereas the rest would not or decide based on the intraoperative assessment.

Involvement of the inferior alveolar nerve is associated with a worse prognosis and requires more extensive resection (54). The question thus addresses the possibility of assessing perineural spread in mandibles with an intact bony cortex. Techniques derived from MR neurography using special sequences, such as 3D double-echo steady-state with water excitation have been shown to have high sensitivity (95–100%) for detection perineural spread (55, 56). While the radiological results have been pathologically validated for HNSCC in general, no publication has specifically targeted the inferior alveolar nerve.

#### Optimal Resection Margins

➢*Adequate resection margin should be 5 mm in T1-2 oral cavity tumors: high LOC (89%)*.

In a T1-2 oral cavity tumor, the adequate resection margin was defined as 5 mm in 8 centers. For one center, it was defined as 10 mm. One center did not provide an answer.

A “sufficient” pathological margin implies a low risk for tumor recurrence and possibly makes adjuvant treatment redundant. However, this issue for oral squamous cell carcinoma is still a subject to debate. Combined analysis (57) of the EORTC 22931 (58) and the RTOG 9501 (59) trials concluded that the adverse prognostic factors requiring adjuvant CRT following surgical resection included extracapsular extension (ECE) of metastatic lymph nodes and positive margins. Somewhat provocative results were published from the Toronto group evaluating oral cavity pN0 patients with margins smaller than 5 mm, treated only surgically: negative margins of 1–5 mm were not associated with inferior local control; while tumor thickness, perineural invasion, and pattern of invasion were predictive of local recurrence (60). The data are in agreement with other studies, establishing pathological scores for resected oral squamous cell carcinoma (61). A review of the literature on the subject seems to confirm that most studies consider 5 mm as a negative margin (60), following the Guideline of the UK Royal College of Pathologists: >5 mm clear, 1–5 mm close, and <1 mm positive margin (62). This discussion pertains to margins assessed by the pathologist and given about 50% shrinkage of the specimen (63), resection should start about 10 mm from the tumor edge.

#### Treatment of Laryngo-Hypopharyngeal Primaries

➢*The status of vocal cord mobility is a key criterion for primary treatment decision: low LOC (63%)*.

In glottic larynx cancer, vocal cord mobility affects the treatment decision in 5/8 centers.

The presence of vocal cord mobility indicates that there is probably an infiltration of the vocal muscle or in rare cases of the crico-arytenoid joint. This is a well-recognized adverse prognostic factor and has been incorporated in the TNM classification for glottic cancer since 1988: an otherwise T1 carcinoma would become a T2 in case of hypomobility, and T3 for complete immobility (64).

The main implication of vocal cord mobility impairment is that the tumor is much bulkier (65) and has extended laterally. Because of this, endoscopic surgery will be more extensive (66) and thus result in more important functional voice and swallowing impairment. Furthermore, especially for T3 cases, the resection might not be possible endoscopically and open partial laryngectomy might become the procedure of choice (67). Even if radiation is the chosen treatment modality, impaired vocal cord mobility carries the main adverse prognostic factor in T2 glottic cancers (68) and is associated with suboptimal cure rates (69).

Why vocal cord mobility does not bring a consensus higher than 63% is difficult to understand. Since vocal cord mobility clearly influences the surgical approach, only possibility is that in some centers, all low stage (T1–T2) carcinoma are treated with radiation therapy and surgeons do not see the mobility as a decisive factor.

➢*Radiologic imaging is reliable to assess laryngeal cartilage invasion: high LOC (86%)*.

Radiologic imaging modalities are considered to be reliable to assess cartilage invasion of larynx cancer in 6/7 centers.

Cartilage invasion has a major impact in the optimal management of laryngeal cancer (see the following question). Cartilage invasion cannot be assessed clinically and therefore, a reliable diagnostic test is essential. The main options are CT and MRI.

It should be kept in mind that the gold standard of evaluating performance of radiological exams is definitive pathology and thus studies evaluating CT and MRI only include patients that underwent surgery which is often total laryngectomy. Thus, compared to the general population of patients with laryngeal cancer, cartilage invasion is probably over-represented, and this bias probably leads to an overestimated positive predictive value (PPV) and to an underestimated negative predictive value (NPV) for the diagnostic modality under investigation.

A recent meta-analysis of CT shows a prevalence of cartilage invasion between 19 and 27%, a PPV ranging between 44 and 80%, and relatively high NPVs ranging between 85 and 100% (70). In other words, false positive CT scans are frequent, while false negative CT scans infrequent and according to the authors, false negative cases stem from minor cartilage invasion, which might not be a contra-indication to conservative treatments, being CRT or partial laryngectomies. Similar results were found in classical studies on the subject (71). However, the performance of CT for extralaryngeal spread is insufficient with NPVs of only 71% (72), making CT not reliable for selecting patients for organ preservation strategies.

MRI can improve the NPVs of CT above 95% in experienced hands (18) and because of its excellent soft tissue evaluation, is the preferred evaluation method for extralaryngeal spread (73). The PPVs are however not better than CT.

➢*To prioritize larynx preservation strategies in cT4a laryngeal primaries or not: no consensus*.

The first choice of treatment in cT4a laryngeal primaries is always to pursue a larynx preservation strategy in one center. Four centers prefer CRT only if the cartilage is not destructed. Other five centers always prefer total laryngectomy followed by adjuvant treatment.

T4a laryngeal carcinoma by definition invades the cartilaginous framework of the larynx and remains best treated by a multimodality regimen, starting with total laryngectomy (74). This has been reemphasized in the recently updated guidelines from the American Society of Clinical Oncology: for “extensive T3 or large T4a lesions and/or poor pretreatment laryngeal function, better survival rates and quality of life may be achieved with total laryngectomy rather than with organ-preservation approaches and may be the preferred treatment strategy” (74).

The debate originated after the VA trial (75) demonstrating that some T4a larynx tumors could be preserved by a CRT protocol. However, in this study, 56% of T4a patients underwent total laryngectomy, especially in glottic primaries with cartilage invasion. Because of that, this population was specifically excluded from the RTOG 91–11 trial (76). This trial was based on the 5th UICC classification of 1992, and the change in the T3–T4 larynx T-staging introduced in the 6th UICC edition added to the confusion. Small inner cortex erosion was classified as T4a in the 5th edition and as T3 in the 6th edition. It is probably safe to say that present day T4 patients were not included in the RTOG 91–11 trial.

As discussed in detail in the guideline of the American Society of Clinical Oncology (74), several high-quality retrospectives studies (77–82) support the better survival of T4a laryngeal cancer patients treated with total laryngectomy, rather than CRT protocols.

➢*The preferred treatment of cT1/2 hypopharyngeal cancer is non-surgical: low LOC (60%)*.

A cT1/2 hypopharyngeal primary is never treated surgically in 6/10 centers.

Hypopharyngeal primaries are associated with low survival (5-year overall survival about 30%) that has barely improved over the years (83). Radiotherapy or CRT are often considered as the standard treatment for hypopharyngeal primaries (84, 85), whereas surgical series with voice preservation are not new (86). No randomized trial has addressed early hypopharyngeal carcinoma. For early T1–T2 primaries, small series with surgical resection, often endoscopic and without adjuvant irradiation, provide encouraging results (Table 2).

**Table 2:** Results of early stage hypopharynx cancer patients in selected surgical series.

**References**	**Stage I (*n*)**	**Stage I-5yLRC**	**Stage II (*n*)**	**Stage II-5yLRC**
Laccourreye (87)			34	95%
Eckel (88)	10	75%	22	75%
Steiner (89)	10	95%	23	95%
Kutter (90)	24	90%	28	90%
Martin (91)	7	73%	19	59%
*K*aratzanis (92)	45	90%	74	83%

*5yLRC: 5-years loco-regional control; n: number*.

## Conclusion

The findings of our survey indicate a low LOC among head and neck oncologists working in academic and multidisciplinary setting in 10 Swiss institutions. Regarding the results and the discussion concerning the specialties other than head and neck surgery, the reader is advised to read the corresponding parts of this article. The highest LOC was achieved among medical oncologists, whereas the lowest was observed among head and neck surgeons. On the other hand, this level of disagreement may also depend on the topics chosen for the survey, and not necessarily the heterogeneity within the disciplines. It is also interesting to witness a low LOC regarding topics, where a high level of evidence actually does exist, and vice versa. This article is expected to serve the head and neck oncologists to be aware of their discrepancies and to stimulate discussion toward standardization of practice and prioritize topics of future clinical research.

## Data Availability Statement

All datasets generated for this study are included in the manuscript/Supplementary Files.

## Author Contributions

GH, MB, OE, PD, and PP: conception and design. OE and PP: collection of data. Generation of the initial and final versions of the questions, drafting of the manuscript, and approval of the final version by all co-authors.

### Conflict of Interest

The authors declare that the research was conducted in the absence of any commercial or financial relationships that could be construed as a potential conflict of interest.
